# Benefits and Risks of Sharing Genomic Data for Research: Comparing the Views of Rare Disease Patients, Informal Carers and Healthcare Professionals

**DOI:** 10.3390/ijerph19148788

**Published:** 2022-07-19

**Authors:** Mariana Amorim, Susana Silva, Helena Machado, Elisa Leão Teles, Maria João Baptista, Tiago Maia, Ngozi Nwebonyi, Cláudia de Freitas

**Affiliations:** 1 Laboratório para a Investigação Integrativa e Translacional em Saúde Populacional (ITR), 4050-600 Porto, Portugal; mariana.f.amorim@gmail.com (M.A.); tiagomtmaia@gmail.com (T.M.); ngozi.nwebonyi@gmail.com (N.N.); 2 EPIUnit—Instituto de Saúde Pública, Universidade do Porto, 4050-600 Porto, Portugal; 3 Centro em Rede de Investigação em Antropologia, Universidade do Minho, 4710-057 Braga, Portugal; susilva08@gmail.com; 4 Instituto de Ciências Sociais, Universidade do Minho, 4710-057 Braga, Portugal; hmachado@ics.uminho.pt; 5 Centro de Referência de Doenças Hereditárias do Metabolismo, Centro Hospitalar Universitário São João, 4200-319 Porto, Portugal; e.leaoteles@gmail.com; 6 Centro de Referência de Cardiopatias Congénitas, Centro Hospitalar Universitário São João, 4200-319 Porto, Portugal; mariajoaobaptista02@gmail.com; 7 Departamento de Ginecologia, Obstetrícia e Pediatria, Faculdade de Medicina, Universidade do Porto, 4200-319 Porto, Portugal; 8 Departamento de Ciências da Saúde Pública e Forenses e Educação Médica, Faculdade de Medicina, Universidade do Porto, 4200-319 Porto, Portugal

**Keywords:** rare diseases, data sharing, genomics research, risks, data governance, public views

## Abstract

Assessing public and patients’ expectations and concerns about genomic data sharing is essential to promote adequate data governance and engagement in rare diseases genomics research. This cross-sectional study compared the views of 159 rare disease patients, 478 informal carers and 63 healthcare professionals in Northern Portugal about the benefits and risks of sharing genomic data for research, and its associated factors. The three participant groups expressed significantly different views. The majority of patients (84.3%) and informal carers (87.4%) selected the discovery of a cure for untreatable diseases as the most important benefit. In contrast, most healthcare professionals revealed a preference for the development of new drugs and treatments (71.4%), which was the second most selected benefit by carers (48.3%), especially by the more educated (OR (95% CI): 1.58 (1.07–2.34)). Lack of security and control over information access and the extraction of information exceeding research objectives were the two most often selected risks by patients (72.6% and 50.3%, respectively) and carers (60.0% and 60.6%, respectively). Conversely, professionals were concerned with genomic data being used to discriminate citizens (68.3%), followed by the extraction of information exceeding research objectives (54.0%). The latter risk was more frequently expressed by more educated carers (OR (95% CI): 1.60 (1.06–2.41)) and less by those with blue-collar (OR (95% CI): 0.44 (0.25–0.77) and other occupations (OR (95% CI): 0.44 (0.26–0.74)). Developing communication strategies and consent approaches tailored to participants’ expectations and needs can benefit the inclusiveness of genomics research that is key for patient-centred care.

## 1. Introduction

Improving genomics research’s potential in the field of rare diseases requires access to large pools of genomic data, biophysical measures, lifestyle information and environment exposures [1,2,3]. However, rare disease patients’ low numbers, geographical dispersal, limited access to quality health care and delayed and/or misdiagnosis make it challenging to gather sufficient data to advance rare disease genomics research [4,5,6,7]. Linking genomic and phenotypic data from the over 800 rare disease registries in Europe and sharing it within and between countries can help to overcome challenges related to data deficits [4,8,9]. The European Reference Networks for rare diseases have received support from the European Commission to gather cross-national rare diseases data into interoperable registries [10] and over sixteen EU countries have signed a declaration to link their genomic databases [11]. These valuable resources can be tapped into by the international research community. However, efforts to promote genomics research also need to take into account data holders’ expectations, concerns and needs. Expanding the evidence base on public and patients’ views about the benefits and risks involved in genomic data sharing is critical to inform the tailoring of research engagement strategies, devise transparent governance frameworks and enable full international collaboration for rare disease genomics research [12,13,14]. Furthermore, harnessing the insight and expertise of patients and their informal carers can contribute to advance patient-centred care by uncovering the need to ensure the translation of genomics research into care that is responsive to patients’ values, preferences and needs from the start [15,16].

Although rare disease patients and their informal carers are generally highly willing to share their data for research, their readiness to engage is subject to a set of data governance requirements [6,17]. Continuous communication, transparent information provision, privacy assurance, consent procedures enabling participants to express preferences about and to retain control over data access, use and reuse, as well as on the return of research results, are key requirements for research engagement [6,17,18,19]. These requirements emerge in response to concerns regarding breaches of privacy and the misappropriation and abuse of information that can lead to the discrimination of people affected by rare diseases by insurance companies or employers [19]. Concerns with data control and ownership were also identified in connection to data sharing with private companies, which may prioritize profit over rare disease patients’ needs [17], and with the reuse of data for purposes that do not align with participants’ values [19]. Nevertheless, these concerns may be trumped by the expected benefits of research [20], which for many people with rare diseases and their informal carers is the only promising pathway to a cure [12].

Public attitudes towards genomic data sharing tend to be shaped by a risk-benefit evaluation that is influenced by a range of factors [1,13,21]. McCormack and colleagues [19] found that both the nature of disease and the sociocultural norms present in different countries impact the attitudes of rare disease patients regarding data sharing. While people affected by life-threatening and progressive rare conditions may feel more lenient about conceding to data (re)use, others whose diseases have a history of stigmatisation (e.g., inherited intellectual disability) may be wary to do so for fearing exclusion from life opportunities should their condition be outrightly identified [19]. Trust in data stewards is another key factor influencing genomic data sharing. Courbier and colleagues [6] identified a “trust gradient” [1] among rare disease patients and their family members in regard to who handles and uses their data. Participants were considerably more likely to trust not-for-profit stakeholders than stakeholders from the for-profit sector. Among the former, they trusted medical doctors involved in their daily care the most, followed by researchers from non-profit organisations, patient organisations and healthcare professionals other than medical doctors [6]. Healthcare professionals thus appear to be in a privileged position to act as gatekeepers and inform patients about genomics studies that might be of particular benefit to them, as well as about their potential risks [22,23]. However, as observed in other fields, healthcare professionals’ views of research benefits and risks may differ from those of patients and their informal carers, particularly when they are not directly involved in conducting research [23].

Most studies to date have focused on the views of rare disease patients and their representatives (e.g., patient advocates and family members) and point to expectations related to genomics research potential to improve diagnosis, enable the development of new drugs and foster the discovery of cures for the various diseases that are still incurable. They also highlight concerns with the need to ensure privacy and adequate procedures to express preferences for data control [17,19,20]. Few studies so far have explored the expectations and concerns of healthcare professionals regarding genomic data sharing [1], and comparative research addressing the views of the various stakeholders involved is lacking. Furthermore, although people’s attitudes towards sharing genomic data are reportedly associated with sociodemographic factors such as age, gender, ethnicity, marital status and educational level [21], these remain under-explored with previous studies suggesting the need to investigate their potential role further [1,2]. In this study, we compare the views of rare disease patients, informal carers and healthcare professionals about the benefits and risks involved in genomic data sharing, and its associated factors, drawing on a cross-sectional study. Our study was carried out in Northern Portugal, which is home to some of the world’s largest clusters of people affected by incurable and stigmatized rare genetic diseases [24,25].

Following previous studies, in this paper, we use the term “genomics research” to generically describe any level of DNA testing, analysis and interpretation [26]. For the purposes of data collection, however, we used the term “genetic information (e.g., DNA)” instead, because the groups under study are more acquainted with this term and it was likely to generate less uncertainty or misunderstandings [2,26].

## 2. Materials and Methods

### 2.1. Participants and Procedure

This cross-sectional study was carried out in the scope of a larger project addressing public and patient involvement in health data governance, whose protocol is published elsewhere [27]. For the purposes of this paper, participants include patients with rare diseases, as well as their informal carers (e.g., family members) and healthcare professionals involved in their care. All participants were recruited at Centro Hospitalar Universitário de São João (CHUSJ), in Porto, Portugal. Patients aged ≥12 years and their informal carers, who attended a consultation between June 2019 and March 2020 at the Reference Centre (RC) for Inherited Metabolic Disorders or the RC for Congenital Heart Diseases from CHUSJ, were consecutively invited to participate in the study. They were handed a study information leaflet by a healthcare professional, after which they were invited to participate by a researcher who clarified any arising questions or doubts about the study. Participants under 18 years old were eligible to participate if they gave verbal consent and their legal representative signed the informed consent form. Those who decided to participate were accompanied to a private setting where they read and signed the informed consent and responded to a self-administered questionnaire. The healthcare professionals who cooperate with the rare diseases RCs’ multidisciplinary team were invited to participate in the study through an e-mail sent by the RCs’ coordinators. Between January and March 2020, a structured questionnaire was distributed to 99 healthcare professionals, along with the informed consent. Healthcare professionals were asked to return the completed questionnaire and the informed consent form in a sealed individual envelope directly to a research team member or by depositing it temporarily in a sealed box placed at their service’s secretariat, according to their preference.

Of the 728 patients and informal carers invited, 77 (23 patients and 54 informal carers) refused to take part in the study due to unwillingness to participate (*n* = 37), lack of time (*n* = 34), lack of consent from the legal tutor (*n* = 3), limited literacy (*n* = 2) and emotional distress following diagnosis (*n* = 1). In total, 651 people (162 patients and 489 informal carers) agreed to participate (response rate: 89.4%). Additionally, 63 healthcare professionals (39 medical doctors, 16 nurses and 8 allied health professionals) completed and returned the questionnaire (response rate: 63.6%).

### 2.2. Data Collection

The research team developed a self-report structured questionnaire based on a literature review and other existing instruments related to the research topic, which is available elsewhere [27]. This questionnaire is the one used for patients and informal carers and it was pretested by five specialists (four women and one man), with combined experience as professionals, informal carers and researchers (social and health sciences), and subsequently piloted by a group of five patients and 22 informal carers between May and June 2019. This process resulted in proposals leading to several linguistic and format modifications, and some items were removed. The questionnaire for healthcare professionals was based on the previous questionnaire and entailed some reformulations in order to capture professionals’ perspectives on the topic. A first version of this questionnaire was tested and reformulated by the coordinators of the two rare diseases RCs, who are medical doctors. The final version comprises information about: (a) positioning about health data use and sharing; (b) opinions about public and patient involvement in health data governance; and (c) sociodemographic characteristics and interpersonal trust.

The risks associated with sharing genetic information were assessed based on the analysis of the following question: “Please select the two most important risks which, in your opinion, can be associated with sharing genetic information for research purposes: (1) Lack of security and control regarding access to information; (2) Restrictions to citizens’ rights of privacy and autonomy; (3) Possibility of extracting information that exceeds the research objectives; (4) Performing genetic studies which can discriminate citizens; (5) Other. Which one?”. The benefits of sharing genetic information were assessed through the following question: “Please select the two most important benefits which, in your opinion, can be associated with sharing genetic information for research purposes: (1) Discovery of a cure for untreatable diseases; (2) Development of strategies to control diseases dissemination; (3) Development of new drugs and treatments; (4) Development of personalised treatments that take into account the characteristics of each patient; (5) Other. Which one?” This study included 700 participants (159 patients, 478 informal carers and 63 healthcare professionals), with data available for the above-mentioned outcomes.

Data on sociodemographic characteristics (sex, age, education, country of origin, marital status, occupation and perceived income adequacy), as well as the participants’ involvement with patients’ organizations and interpersonal trust, was collected. Patients and carers also reported their level of satisfaction with own health. Occupations were classified according to the Portuguese Classification of Occupations 2010 [28] and grouped into four categories: (1) upper-white-collar, including executive civil servants, industrial directors and executives, professionals and scientists, middle management and technicians; (2) lower-white-collar, including administrative and related workers, service and sales workers; (3) blue-collar, which includes farmers and skilled agricultural workers, fisheries workers, skilled workers, craftsmen and similar, machine operators and assembly workers, unskilled workers; and (4) other, including students (*n* = 122), those unemployed (*n* = 78), participants doing housework (*n* = 26), participants who are pensioners or in a paid/unpaid leave (*n* = 12), retired (*n* = 11) and informal carers or members of a foster family (*n* = 5). Perceived income adequacy was measured through the question “When thinking of your household income, would you say that your household is able to make ends meet?”. Participants could check one of the following answer categories: insufficient, caution with expenses, enough to make ends meet and comfortable.

Patients and informal carers were also asked about their willingness to share genetic information (e.g., DNA) for research purposes, using a five-point Likert scale ranging from 0 (not willing) to 4 (always willing). For the purpose of this study, we dichotomized this variable into willing/ always willing (3 and 4) and other (0, 1 and 2).

### 2.3. Data Analysis

Participants’ characteristics are presented as counts and proportions for categorical variables (sex, age, educational level, country of origin, marital status, occupation, perceived income adequacy, involvement in patient organisations, satisfaction with own health and willingness to share genetic information for research). A Shapiro–Wilk test was performed and showed that the distribution of interpersonal trust departed significantly from normality (W = 0.982, *p*-value < 0.001); thus, this non-normally distributed continuous variable is described as median and interquartile range (P25–P75).

The prevalence of the outcomes (the two most important benefits and risks of sharing genetic information for research) is presented as count and proportions, stratified by type of participant. The Chi-square test was used to compare the outcomes between the participant groups (patients, informal carers and healthcare professionals).

The associations between explanatory variables and the most frequently selected benefits and risks of sharing genetic information for research were assessed through the Chi-square test and the Mann–Whitney test for categorical variables and the interpersonal trust, respectively (data not shown). All variables with a *p* value < 0.05 were included in the logistic regression models, stratified by type of participant, to estimate the odds ratios (ORs) and corresponding 95% confidence intervals (95% CI) for the association between the sociodemographic characteristics, and the willingness to share genetic information for research purposes and the most relevant benefits and risks. The final model for benefits included sex, age, educational level, occupation and perceived income adequacy, whereas the final model for risks included sex, educational level, occupation and willingness to share genetic information for research.

The statistical analysis was performed using the software IBM SPSS Statistics for Windows, version 25.0 (IBM Corp., Armonk, NY, USA).

## 3. Results

The characteristics of patients, informal carers and healthcare professionals, as well as patients’ and informal carers’ willingness to share genetic information for research, are shown in Table 1. The majority of the informal carers (80%) and healthcare professionals (67%) were female, while 53% of the patients were male. Informal carers and healthcare professionals were more frequently married or living with a partner than patients (77.9% and 79.4%, respectively, vs. 10.1%) and had more upper white-collar occupations (32.3% and 100%, respectively, vs. 3.2%). More than half of informal carers perceived their income as insufficient (56.4%), while most patients (64.6%) and healthcare professionals (80.6%) considered it comfortable/enough to make ends meet. Only 6.1% of informal carers, 3.2% of healthcare professionals and 3.1% of patients were born in a country other than Portugal. Informal carers reported more frequently that they were satisfied or very satisfied with their own health than patients (76.3% vs. 62.3%). Healthcare professionals (17.7%) were more frequently involved in patient organisations compared to informal carers and patients (6.3% vs. 1.9%, respectively). Patients and informal carers presented lower levels of interpersonal trust (Median [P25–P75]: 4.7 [2.5–6.7)] and Median [P25–P75]: 4.7 [3.0–6.3], respectively) than healthcare professionals (Median [P25–P75]: 6.0 [3.7–7.7]. Patients and informal carers revealed to be similarly willing to share genetic information for research (70.3% vs. 67.9%, respectively).

### 3.1. Benefits and Risks of Sharing Genetic Information

The three participant groups expressed significantly different views on the two benefits and risks most prominently associated with genetic information sharing for research purposes (Table 2). The great majority of patients (84.3%) and informal carers (87.4%) selected the possibility of discovering a cure for untreatable diseases as one of the most important benefits. In contrast, most healthcare professionals revealed a preference for the benefit of developing new drugs and treatments (71.4%), which was the second and third most selected benefit by informal carers (48.3%) and patients (39.0%), respectively. The development of personalised treatments that take into account the characteristics of each patient constituted the third most selected benefit by informal carers (37.2%) and healthcare professionals (33.3%), but the second by patients (42.8%). Finally, the development of strategies to control disease dissemination was considered an important benefit by less than one third of patients (32.1%), healthcare professionals (30.2%) and informal carers (25.1%).

Regarding the risks associated with sharing genetic information for research, the lack of security and control over access to information and the possibility of extracting information that exceeds the research objectives were the two most often selected risks by patients (72.6% and 50.3%, respectively) and informal carers (60.0% and 60.6%, respectively) (Table 2). In contrast, healthcare professionals were especially concerned with the risk of performing genetic studies that can be used to discriminate citizens (68.3%), as well as with the possibility of extraction of information that exceeds the research objectives (54.0%). The potential risk of restrictions to citizens’ privacy and autonomy was selected by 40.8% of patients, 33.1% of informal carers and 27% of healthcare professionals.

### 3.2. Factors Associated with the Selection of Benefits and Risks of Sharing Genetic Information

Informal carers with more than 12 years of education (OR (95% CI): 2.20 (1.11–4.36)) and who perceived their income as enough to make ends meet or comfortable (OR (95% CI): 2.08 (1.14–3.77)) revealed a statistically significant tendency to select the discovery of a cure for untreatable diseases as a major benefit, while informal carers with blue-collar occupations were less likely to select it (OR (95% CI): 0.31 (0.14–0.68)) (Table 3). Healthcare professionals who were 49 years of age or above (OR (95% CI): 3.29 (1.04–10.41)) and with better perceived income (OR (95% CI): 5.60 (1.47–21.40)) selected more frequently the development of new drugs and treatments as a major benefit, as did informal carers with higher levels of education (OR (95% CI): 1.58 (1.07–2.34)).

Informal carers who were more willing to share genetic information (OR (95% CI): 0.62 (0.41–0.93)) were less likely to select the risk of lack of security and control regarding access to information (Table 4). The possibility of extracting information exceeding the research objectives was stated more frequently by informal carers with higher levels of education (OR (95% CI): 1.60 (1.06–2.41)), while those with blue-collar and other occupations were less likely to select that risk (OR (95% CI) blue-collar: 0.44 (0.25–0.77; OR (95% CI) other occupations: 0.44 (0.26–0.74)). Patients and informal carers who were more willing to share genetic information were also more worried about the risk of performing genetic studies that can be used to discriminate citizens (OR (95% CI) patients: 2.86 (1.25–6.52); OR (95% CI) informal carers: 2.04 (1.35–3.10)).

## 4. Discussion

The rare disease patients, informal carers and healthcare professionals surveyed differed significantly in their views about the benefits and risks of sharing genomic data for research. These differences were also associated with several factors including age, educational level, occupation, income and willingness to share genomic data. Importantly, although the majority of patients and informal carers were more willing to share genetic information for research (70% and 67%, respectively) than the general Portuguese population (56%) [29], the overall proportion of participants who were willing to share was substantially lower than that identified in a study carried out under the EURORDIS Rare Barometer Programme (97%) [6]. Comparing the benefits and risks of sharing genomic data considered most relevant by the three participant groups may help us to understand these disparities, as well as to provide insights to design and implement genomics research policy proportional to individuals’ expectations and concerns [14].

Patients and informal carers valued significantly more the discovery of a cure for untreatable diseases as a potential benefit of sharing genomic data for research than healthcare professionals. Furthermore, informal carers and healthcare professionals were more likely to select the prospect of genomics research resulting in the development of new drugs and treatments, while patients valued its potential to foster the delivery of personalised treatments. The differences identified contrast with the results of previous studies that point to patients, family members and other members of the public having similar perspectives about the benefits they expect to derive from sharing health data (e.g., advancements in healthcare through enhanced diagnosis and treatment options) [13,17,21]. Nevertheless, the patients’ focus on improved individual health outcomes in exchange for data sharing has been consistently reported in the literature [21,30].

For people suffering from a rare disease, benefiting from personalised treatments that take into consideration their individual genetic variability, environment exposures and lifestyle is a legitimate and expectable aspiration. Some patients spend a large part of their lives without a diagnosis, or are misdiagnosed, which impedes them from accessing appropriate care [7,31,32,33]. The hope lent by genomics research to predict disease and introduce molecular therapies tailored to each patients’ specific needs [34] may help to explain why patients in our study perceived the development of personalised treatments as one of its most prominent benefits. However, the translation of genomic knowledge into treatments for inherited diseases has been slow [34]. Furthermore, progress in the implementation of personalised therapies has been uneven across the globe both due to limited evidence demonstrating its clinical utility and lack of investment in innovation [35]. Awareness of these circumstances may have led healthcare professionals in our study to be less prone to select personalised treatments when compared to patients, and instead elect the development of new drugs and treatments as the benefit most likely to emerge from genomics research. Such drugs and treatments can help to reduce or halt the progression of disease and may appear as more viable attainments. This view may be shared by informal carers who also elected this as one of the most salient benefits.

Given rare disease patients’ and informal carers’ high level of trust in healthcare professionals [6], and their often key role in research recruitment, it would be important to offer training in genomics to healthcare professionals [36]. This will likely help to clarify what are reasonable expectations from genomics research, thus rendering communication between all parties more fluid, transparent and dependable. Genomics research will not necessarily translate into the delivery of individual therapeutic benefits in the short-run, but rather into a set of discoveries that may impact patients’ personal health outcomes in the future [37,38,39]. Nevertheless, access to this information is unlikely to discourage patients and their informal carers from sharing genomic data. Several studies show that people affected by rare diseases are committed to promoting the greater good and would share their data even if that is tied with delivering benefits only to upcoming generations [6,17,40]. Providing accurate and comprehensible information about the benefits, risks and procedures of genomics to patients and informal carers is thus essential to empower them to make informed decisions about data sharing and to prevent thwarted expectations and research withdrawal [36,41].

Participants in our study held significantly different views about the risks most prominently associated with genomic data sharing, similarly to previous studies [13]. While patients and informal carers showed greater concerns with lack of security and control regarding access to information, healthcare professionals were more worried about the potential for genomics research to be used to discriminate patients and their families. Concerns about data security and control were expressed more frequently by informal carers who were less willing to share genomic data. To a certain extent, these concerns may explain informal carers’ lower disposition to share data when compared to other members of the international rare diseases community [6]. Concerns about genetic discrimination were expressed more frequently by older healthcare professionals with higher incomes who, in our sample, are more likely to match the profile of medical doctors. These concerns may originate on their lived experience of caring for people with and at risk for rare genetic diseases who, as the literature shows, are fearful of genomic information being misused by insurance companies or employers to discriminate them [12,42,43,44,45]. Fears of genetic discrimination are often rooted in previous experiences of disease-related stigmatisation and discrimination within the family, or in social settings, and may lead people to abstain from genetic testing, which jeopardises their access to care [46].

Offering genomics research participants more direct control over who accesses their data can help to reduce concerns with data security and discriminatory practices [17,19]. In a previous study with the same patient and informal carer populations, we found that the majority of participants valued being involved in decision-making about data access (91% of carers and 75% of patients) [18]. Enabling people affected by rare diseases to express their preferences about data management by offering them an option to engage through dynamic consent, as suggested by several scholars [47,48], can contribute to both satisfy their expectations for greater involvement and increase transparency and accountability in data governance processes [3,49]. Dynamic consent approaches rely on online platforms to facilitate a two-way ongoing communication between researchers and participants [47,48,50]. In addition to allowing participants to tailor and change their data preferences over time, online platforms can also be used to inform participants about the research progress, request permission for data access by third parties and ask participants to upload new data [47,51]. These interactions can increase participants’ sense of control and security by building and maintaining their trust in genomics researchers and data governance practices [3]. However, involvement through dynamic consent demands a specific set of resources (e.g., time, information, digital literacy and access to digital devices), which are not equally available to all potential participants and that may discourage those least resourced from participating. To prevent this type of consent from unintendedly reinforcing inequalities, it is necessary to ask participants about their preferred modes for consent and to support them in realising their preferences by providing information about the benefits and risks of genomics research, using innovative methodologies (e.g., arts-based methods), facilitating access to digital technologies and offering data counselling when needed [52,53,54,55]. A meta consent model [56] may therefore be a more suitable approach, particularly in settings in which these conditions are not yet met. Meta consent enables research participants to select their preferred consent mode from a range of options (i.e., blanket, broad and dynamic consent and blanket refusal) at a given time, while keeping open the possibility to shift preference to another consent mode in the future. This approach not only affords participants more agency in acquiring and managing the resources needed for ongoing involvement in decision-making, it can also help to avoid consent fatigue [56], particularly at times when disease presentation is acute and harder to manage for both patients and their families.

The sociodemographic factors influencing participants’ views about the benefits and risks of genomics data sharing included education, occupation and income, and were especially salient among informal carers. Informal carers with higher levels of education and better incomes expressed greater hope in genomics research’s potential to enable the discovery of a cure for the various rare diseases that remain untreatable. They were also significantly more likely to be worried with the risk of information being extracted and used beyond the original purposes of research which, as some studies show, relate to the concern of genomic data being linked to personal details and used by government or for commercial purposes (e.g., the government acquiring information that people do not intend to share; being targeted by marketing companies for sales) [6,53]. People with favourable socioeconomic positions may enjoy greater awareness about the implications of genomics research and, as a result, be more prone to identify both its revolutionary potential and its potential hazards. Conversely, less advantaged groups may experience more information needs and have less resources to discern between potential risks. An earlier study involving predominantly lower income ethnically diverse communities found that most participants had a limited understanding of genetics research and only a minority expressed major concerns about genetic testing [57]. Given the complexity involved in genomics research, and the positive effects of increased awareness on individuals’ intention to share genomic data [21,34,58], it would be important to inquire further about the information needs of people affected by rare diseases in Portugal and to design communication strategies and public campaigns tailored to meeting those needs, with particular attention to socially disadvantaged individuals.

Restrictions to citizens’ rights to privacy and autonomy that may unfold as a result of data breaches was the least often selected risk by informal carers and healthcare professionals, while patients perceived it to be slightly more worrisome. This finding contrasts with the existing literature which has identified loss of privacy as a key concern of people involved in genomics research [13,21,59]. A possible explanation may relate to trust in science, which is relatively high among the Portuguese population [60]. Patients and informal carers may have felt that research endeavours are generally trustworthy and that researchers will reciprocate the trust involved in the act of sharing genomic data by protecting their identity and guaranteeing their privacy [61]. However, they may have also considered that identifiability is minor risk that they are willing to bare to advance scientific progress and assist others [62]. Furthermore, patients often choose for re-identification to enable the return of research results, which precludes anonymization [62,63]. These are, nevertheless, tentative explanations that should be taken with caution. Having a diagnosis for an incurable rare disease places patients and their families in a position of vulnerability that may impel them to relinquish the protection of their privacy in exchange for the prospect of research delivering a cure [6,12,19]. Thus, as argued by Mascalzoni and colleagues [64], requests to share genomic data for research based on solidarity towards one’s kin, other patients or society at large have to be balanced against the need to protect patients’ privacy and autonomy, as well as their relatives’.

### Strengths and Limitations

This is one of a few studies comparing the views of rare disease patients, informal carers and healthcare professionals about the most salient benefits and risks of genomic data sharing for research, and examining its association with willingness to share genomic information and sociodemographic variables. A key contribute of this study has been to increase evidence on the similarities and differences in patients’, informal carers’ and healthcare professionals’ views of the benefits and risks of genomic data sharing. Eliciting the views and expertise of patients and their carers and incorporating it into practice is at the core of the patient-centred care paradigm [16]. By comparing the views of the various stakeholders involved in genomics research, this paper emphasises the need to realise the importance of identifying and integrating their values, preferences and expressed needs not only in research engagement and governance practices but also throughout the process of translation of genomics research into clinical practice.

Participants were consecutively invited to participate at two reference centres for rare diseases located at an academic hospital centre, which is a referral hospital for a total of 3.5 million people in Portugal’s northern region. Nonetheless, recruitment in one single region limits the generalizability of the results to the whole population. Furthermore, there is an overrepresentation of young patients in our sample. Recruitment initiated at services that care mostly for paediatric patients, many of whom were below the age of 12 and were accompanied by their informal carers. It subsequently ensued to services caring for adults but that occurred shortly before the start of the COVID-19 pandemic upon which data collection had to be discontinued. The proportion of informal carers is thus larger than the proportion of participants in the other two groups, which may explain part of the significant associations with sociodemographic factors that were found. The number of patients and carers who were involved in patient organisations is substantially lower (1.9% and 6.2%, respectively) than that found in studies carried out with people affected by rare diseases elsewhere (66%) [17]. Although the proportion of participants involved reflects the relative low number and uneven distribution of patient organisations across the Portuguese territory [65], direct comparisons with previous studies need to be observed with caution. This study is suggestive of the possible factors associated with the assessment of the benefits and risks of sharing genetic information for research at the time of data collection. However, it not possible to establish a temporal relationship. Finally, future research would benefit from using qualitative methods to understand the reasons and motivations underlying the perspectives of the various stakeholder groups.

## 5. Conclusions

Genomics research researchers need to consider the diversity of views held by rare disease patients, informal carers and healthcare professionals when discussing the benefits and risks of sharing genomic data. Such diversity may derive from distinct levels of access to information about and understanding of the implications of genomics research and data sharing. Thus, in addition to tailoring communication strategies to participants’ expectations and needs, it would be important to consider the adoption of consent approaches that enable participants to express their preferences about the degree of involvement and control they wish to have when making decisions about who accesses their data and for what purposes. This will likely contribute to promote adequate data governance and increase the inclusiveness of rare diseases genomics research, which is essential to develop care centred on patients’ needs.

## Figures and Tables

**Table 1:** Participants’ characteristics and willingness to share genetic information for research.

	Patients (*n* = 159)	Informal Carers (*n* = 478)	Healthcare Professionals (*n* = 63) ^a^
**Sex**			
Female	75 (47.2)	376 (78.7)	42 (66.7)
Male	84 (52.8)	102 (21.3)	21 (33.3)
**Age (years)**			
<18	89 (56.0)	0 (0)	0 (0)
18–29	44 (27.7)	47 (9.9)	0 (0)
30–49	20 (12.6)	356 (75.1)	29 (46.0)
>49	6 (3.8)	71 (15.0)	34 (54.0)
**Educational level (years)**			
≤12	151 (95.6)	327 (68.8)	0 (0)
>12	7 (4.4)	148 (31.2)	63 (100)
**Country of origin**			
Portugal	154 (96.9)	447 (93.9)	61 (96.8)
Other ^b^	5 (3.1)	29 (6.1)	2 (3.2)
**Marital status**			
Married/living with partner	16 (10.1)	371 (77.9)	50 (79.4)
Other ^c^	142 (89.9)	105 (22.1)	13 (20.6)
**Occupation**			
Upper white-collar	5 (3.2)	144 (32.3)	63 (100)
Lower white-collar	7 (4.4)	108 (24.2)	0 (0)
Blue-collar	9 (5.7)	79 (17.7)	0 (0)
Other ^d^	137 (86.7)	115 (25.8)	0 (0)
**Perceived income adequacy**			
Insufficient/caution with expenses	51 (35.4)	267 (56.4)	12 (19.4)
Enough to make ends meet/comfortable	93 (64.6)	206 (43.6)	50 (80.6)
**Involvement in patient organisations**			
No	154 (98.1)	446 (93.7)	51 (82.3)
Yes	3 (1.9)	30 (6.3)	11 (17.7)
**Satisfaction with own health**			
Very unsatisfied/Unsatisfied	16 (10.1)	34 (7.1)	
Neither satisfied nor unsatisfied	44 (27.7)	79 (16.6)	--
Satisfied/Very satisfied	99 (62.3)	364 (76.3)	
**Willingness to share genetic information for research**			
Always willing/willing	109 (70.3)	320 (67.9)	--
Other	46 (29.7)	151 (32.1)	--
**Interpersonal Trust, Md (P25–P75)**	4.7 (2.5–6.7)	4.7 (3.0–6.3)	6.0 (3.7–7.7)

^a^ includes physicians (*n* = 39), nurses (*n* = 16) and allied health professionals (*n* = 8). ^b^ includes Brazil (*n* = 9), France (n = 7), Angola (*n* = 5), Venezuela (*n* = 3), Mozambique (*n* = 3), Cape Verde (*n* = 2), Germany (*n* = 2), United Kingdom (*n* = 1), Switzerland (*n* = 1), São Tomé e Príncipe (*n* = 1) and South Africa (*n* = 1). One carer did not report the country of origin. ^c^ includes single (*n* = 204), widowed (*n* = 6), divorced (*n* = 42) and separated (married, but does not live with partner) participant (*n* = 4). ^d^ includes unemployed (*n* = 77), retired (*n* = 11), participants doing housework (*n* = 25), informal carers or members of a foster family (*n* = 5), participants who are pensioners or in a paid/unpaid leave (*n* = 12) and students (*n* = 122). Notes: Values are presented as count and proportions unless otherwise specified; in each variable, the total may not add 159 patients, 478 informal carers and 63 healthcare professionals due to missing values; the pro-portions may not add 100 due to rounding; Md—Median.

**Table 2:** Participants’ views about the two most important benefits and risks of sharing genetic information for research.

Benefits	Patients (*n* = 159)	Informal Carers (*n* = 478)	Healthcare Professionals (*n* = 63)	*p* Value
Discovery of a cure for untreatable diseases	134 (84.3)	418 (87.4)	40 (63.5)	**<0.001**
Development of new drugs and treatments	62 (39.0)	231 (48.3)	45 (71.4)	**<0.001**
Development of personalised treatments, taking into account the characteristics of each patient	68 (42.8)	178 (37.2)	21 (33.3)	0.329
Development of strategies to control disease dissemination	51 (32.1)	120 (25.1)	19 (30.2)	0.197
Other: Help other people ^a^	0 (0)	1 (0.2)	0 (0)	NA
**Risks**	**Patients** **(*n* = 157)**	**Informal** **Carers** **(*n* = 477)**	**Healthcare** **Professionals** **(*n* = 63)**	***p* Value**
Lack of security and control over access to information	114 (72.6)	286 (60.0)	31 (49.2)	**0.002**
Possibility of extracting information that exceeds the research objectives	79 (50.3)	289 (60.6)	34 (54.0)	0.064
Performing genetic studies that can discriminate citizens	54 (34.4)	189 (39.6)	43 (68.3)	**<0.001**
Restrictions to citizens’ rights of privacy and autonomy	64 (40.8)	158 (33.1)	17 (27.0)	0.095
Other: Misuse of information ^a^	0 (0)	2 (0.4)	0 (0)	NA
Other: Commercialisation of information ^a^	0 (0)	1 (0.2)	0 (0)	NA

^a^ Answers reported by the participants using the open-ended option. Notes: Participants were asked to select the two most important risks and benefits; Values are presented as count and proportions; Several answers possible, so percentage does not total 100%; Bold types represent statistical significance.

**Table 3:** Factors associated with selecting the benefits of sharing genetic information for research, stratified by type of participant.

	Discover a Cure for Untreatable Diseases Crude OR (95% CI)			Development of New Drugs and Treatments Crude OR (95% CI)			Development of Personalised Treatments Crude OR (95% CI)		
	Patients	Informal Carers	Healthcare Professionals	Patients	Informal Carers	Healthcare Professionals	Patients	Informal Carers	Healthcare Professionals
**Sex**									
Female	1	1	1	1	1	1	1	1	1
Male	1.04 (0.44–2.45)	1.10 (0.56–2.15)	0.49 (0.17–1.45)	0.92 (0.49–1.75)	0.77 (0.49–1.19)	1.43 (0.43–4.76)	1.01 (0.54–1.89)	0.72 (0.45–1.15)	1.00 (0.33–3.04)
**Age (years)**									
<18	1	--	--	1	--	--	1	--	--
18–29	0.90 (0.33–2.46)	1	--	1.18 (0.56–2.46)	1	--	0.74 (0.35–1.54)	1	--
30–49	0.68 (0.20–2.37)	2.08 (0.96–49)	1	1.39 (0.52–3.70)	0.58 (0.31–1.08)	1	0.63 (0.23–1.73)	1.02 (0.55–1.91)	1
>49	0.86 (0.09–7.92)	1.86 (0.69–5.00)	0.64 (0.23–1.82)	0.58 (0.15–4.89)	0.70 (0.33–1.47)	**3.29 (1.04–10.41)**	1.17 (0.22–6.12)	0.68 (0.31–1.47)	0.38 (0.13–1.11)
**Educational level (years)**									
≤12	1	1	--	1	1	--	1	1	--
>12	1.08 (0.12–9.38)	**2.20 (1.11–4.36)**		1.17 (0.25–5.41)	**1.58 (1.07–2.34)**		0.22 (0.03–1.83)	0.84 (0.56–1.26)	
**Occupation**									
Upper white-collar	1	1	--	1	1	--	--	1	--
Lower white-collar	1.50 (0.07–31.58)	1.00 (0.41–2.47)		2.00 (0.19–20.61)	0.95 (0.57–1.56)			0.92 (0.55–1.56)	
Blue-collar	0.31 (0.02–4.02)	**0.31 (0.14–0.68)**		1.88 (0.20–17.27)	0.68 (0.39–1.18)			1.21 (0.69–2.12)	
Other ^a^	1.65 (0.18–15.63)	0.49 (0.23–1.06)		0.86 (0.14–5.34)	0.87 (0.53–1.42)			1.10 (0.66–1.82)	
**Perceived income**									
Insufficient/caution with expenses	1	1	1	1	1	1	1	1	1
Enough to make ends meet/comfortable	1.32 (0.52–3.34)	**2.08 (1.14–3.77)**	0.82 (0.22–3.08)	0.90 (0.45–1.81)	1.16 (0.81–1.67)	**5.60 (1.47–21.40)**	1.28 (0.64–2.56)	0.89 (0.61–1.30)	0.43 (0.12-1.55)

^a^ includes unemployed, retired, participants doing housework, informal carers or members of a foster family, participants who are pensioners or in a paid/unpaid leave and students. Bold types represent statistical significance.

**Table 4:** Factors associated with selecting the risks of sharing genetic information for research, stratified by type of participant.

	Lack of Security and Control Over Access to Information Crude OR (95% CI)			Possibility of Extracting Information That Exceeds the Research Objectives Crude OR (95% CI)			Performing Genetic Studies That Can Discriminate Citizens Crude OR (95% CI)		
	Patients	Informal Carers	Healthcare Professionals	Patients	Informal Carers	Healthcare Professionals	Patients	Informal Carers	Healthcare Professionals
**Sex**									
Female	1	1	1	1	1	1	1	1	1
Male	0.85 (0.42–1.72)	1.16 (0.74–1.82)	0.68 (0.24–1.96)	1.02 (0.55–1.92)	1.01 (0.65–1.58)	1.21 (0.42–3.48)	0.75 (0.39–1.45)	1.20 (0.77–1.88)	1.25 (0.40–3.92)
**Educational level**									
≤12	1	1	--	1	1	--	1	1	--
>12	0.92 (0.17–4.92)	1.03 (0.69–1.53)		0.72 (0.16–3.33)	**1.60 (1.06–2.41)**		0.77 (0.14–4.10)	0.85 (0.57–1.26)	
**Occupation**									
Upper white-collar	1	1	--	1	1	--	1	1	--
Lower white-collar	3.33 (0.20–54.53)	1.03 (0.62–1.72)		8.00 (0.50–127.90)	0.65 (0.38–1.12)		2.00 (0.13–31.98)	1.12 (0.67–1.87)	
Blue-collar	0.33 (0.04-3.21)	0.91 (0.52–1.60)		5.00 (0.39–64.39)	**0.44 (0.25–0.77)**		2.00 (0.15–26.73)	1.61 (0.92–2.81)	
Other ^a^	2.00 (0.32–12.48)	0.97 (0.59–1.60)		4.12 (0.45–37.81)	**0.44 (0.26–0.74)**		2.18 (0.24–20.08)	1.23 (0.74–2.05)	
**Willingness to share genetic data**									
Other	1	1	--	1	1	--	1	1	--
Always willing/willing	0.54 (0.24–1.26)	**0.62 (0.41–0.93)**		0.96 (0.48–1.92)	1.00 (0.67–1.48)		**2.86 (1.25–6.52)**	**2.04 (1.35–3.10)**	

^a^ includes unemployed, retired, participants doing housework, informal carers or members of a foster family, participants who are pensioners or in a paid/unpaid leave and students. Bold types represent statistical significance.

## Data Availability

The data presented in this study are available on request from the corresponding author. The data are not publicly available due to a confidentiality agreement securing participants’ privacy and anonymity.
